# Targeted Transvenous Embolization of Cavernous Sinus Dural Arteriovenous Fistula With Liquid Materials Using a Dual-Lumen Balloon Microcatheter

**DOI:** 10.7759/cureus.13821

**Published:** 2021-03-11

**Authors:** Yosuke Kawamura, Tomoji Takigawa, Akio Hyodo, Kensuke Suzuki

**Affiliations:** 1 Neurosurgery, Dokkyo Medical University Saitama Medical Center, Koshigaya, JPN

**Keywords:** cavernous sinus dural arteriovenous fistula, dual-lumen balloon microcatheter, liquid materials, targeted transvenous embolization

## Abstract

We describe a challenging transvenous embolization technique involving a dual-lumen balloon microcatheter (DLBM) and liquid materials for cavernous sinus dural arteriovenous fistula (CSDAVF).

DLBM contributed to identifying the shunt point and preventing liquid material leakage to normal venous drainage without treatment-related complications. In a transvenous embolization using liquid materials for CSDAVF complications such as cranial nerve palsy and embolic agent migration into the internal carotid artery due to numerous anastomoses must be considered. The use of angiography during DLBM inflation to characterize the shunt point and DLBM to prevent liquid material leakage to the normal venous drainage might decrease the mass effect due to liquid materials, thereby minimizing the causes of newly occurring cranial nerve palsy.

This technique may be helpful for the treatment of CSDAVF in practice, but there is generally a risk in using liquid materials in the cavernous sinus; therefore, further consideration is needed in the future.

## Introduction

The natural course of a cavernous sinus dural arteriovenous fistula (CSDAVF) is more benign than that of a dural arteriovenous fistula (DAVF) arising at other sites. These lesions regress spontaneously in 5.3% of cases [1]. However, curative treatment should be administered for a CSDAVF associated with visual disturbances and cortical venous reflux [2], and treatments are predominantly endovascular. The gold standard treatment is transvenous embolization (TVE) with coils, ideally with a target comprising only the fistulous point [3,4].

Onyx™ (Medtronic, Minneapolis, MN, USA) is a liquid mixture of an ethylene vinyl alcohol copolymer suspended in dimethyl sulfoxide (DMSO) and tantalum powder. This non-adhesive liquid exhibits excellent penetration of complex vascular structures, allows user-based control to reduce the risk of accidental embolization, and can be delivered through a single slow injection [5]. Transvenous Onyx injection for an indirect carotid-cavernous fistula was first described in 2004 [6]. Although transvenous Onyx embolization is the current mainstay of treatment for indirect carotid-cavernous fistulas, it has only been reported in small case series of DAVF [5,7]. Target coil embolization is an effective and safe method that does not exacerbate symptoms [8], and coil deployment before Onyx injection slows the fistula flow and securely anchors the Onyx cast [9].

Although transarterial embolization (TAE) with Onyx has been reported [10,11], its use is limited by anatomical features and the possibility of cranial nerve dysfunction [12]. Use of transarterial balloon-assisted embolization with Onyx and dual-lumen microcatheters for aggressive DAVF has also been reported [4]. A compliant balloon might be navigated into the transverse sinus to protect the normal sinus from Onyx reflux in a DAVF of the transverse-sigmoid sinus [13]. However, TVE with liquid materials and a dual-lumen balloon microcatheter (DLBM) is not a well-established technique for CSDAVF. Here, we describe the useful technique of TVE with liquid materials and a DLBM for CSDAVF.

## Technical report

We searched a prospectively maintained single-center database for patients who underwent CSDAVF treatment between December 2009 and 2019. All patients were examined in our hospital by an independent neurosurgeon who examined their eyes and measured neurological outcomes using the modified Rankin Scale (mRS), ocular symptoms, and cranial nerve palsy (cranial nerves III, IV, V, or VI). Four-vessel digital subtraction angiography was performed before and immediately after embolization and at the six-month follow-up. Some patients underwent magnetic resonance angiography at the 6- and 12-month follow-ups.

The safety end point was the incidence of treatment-related complications, which were defined as vessel perforations during embolization, hemorrhage or ischemic stroke, death, asystole, or new neurologic or ocular deficits. The efficacy end points were complete angiographic occlusion at 12 months after treatment and improvements in ocular symptoms and cranial nerve palsy at 12 months after treatment.

All procedures were performed under general anesthesia, and continuous anticoagulation was maintained with heparin sodium. Of the two embolization strategies, venous embolization was usually the first treatment choice, whereas the arterial approach was indicated if venous embolization could not be achieved. For TVE, the fistula compartment of the cavernous sinus was catheterized through the inferior petrosal sinus (IPS). When embolic materials were used, coiling by venous access was first indicated. Liquid materials were injected through venous access into the cavernous sinus when complete fistula occlusion could not be achieved with coils alone.

Onyx was approved in Japan for DAVF treatment on April 25, 2018. However, the following statement was included in the attached document about Onyx: “unless the doctors decide that it is unavoidable, the doctors should not perform with Onyx in the cavernous sinus” [14]. Our hospital’s Institutional Review Board approved the use of Onyx to achieve complete CSDAVF occlusion. Recently, we injected n-butyl-2-cyanoacrylate (NBCA, glue, TRUFILL®, DePuy Synthes, Raynham, MA, USA) into fistulas restricted to the small cavernous sinus compartment after coil embolization without Onyx. The use of DLBMs (Scepter XC™, MicroVention, Tustin, CA, USA) was approved in Japan on March 25, 2013, and they were subsequently used to determine the precise location of the shunt and manage liquid materials.

**Figure 1:**
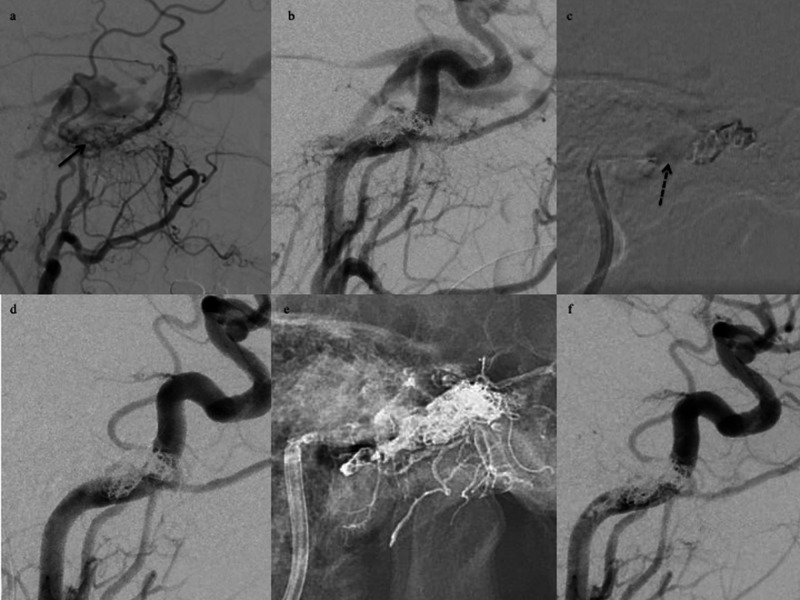
Illustrative case 1. A DLBM was used to identify the shunt point. Left external carotid artery angiograms. (a) Lateral view, arterial phase. Angiograms reveal the fistulous shunt in the posteromedial compartment of cavernous sinus (black arrows). (b) A post-coil embolization CCAG reveals non-occlusion of the shunt. (c) A dual-lumen balloon microcatheter inflation (dotted black arrow) is revealed on the road map. (d) An angiogram during balloon inflation reveals occlusion of the residual fistula. (e) The cast of Onyx is visible at the end of the injection. (f) A post-embolization CCAG reveals non-opacification of the fistulous shunt. DLBM, dual-lumen balloon microcatheter; CCAG, common carotid artery angiogram

**Figure 2:**
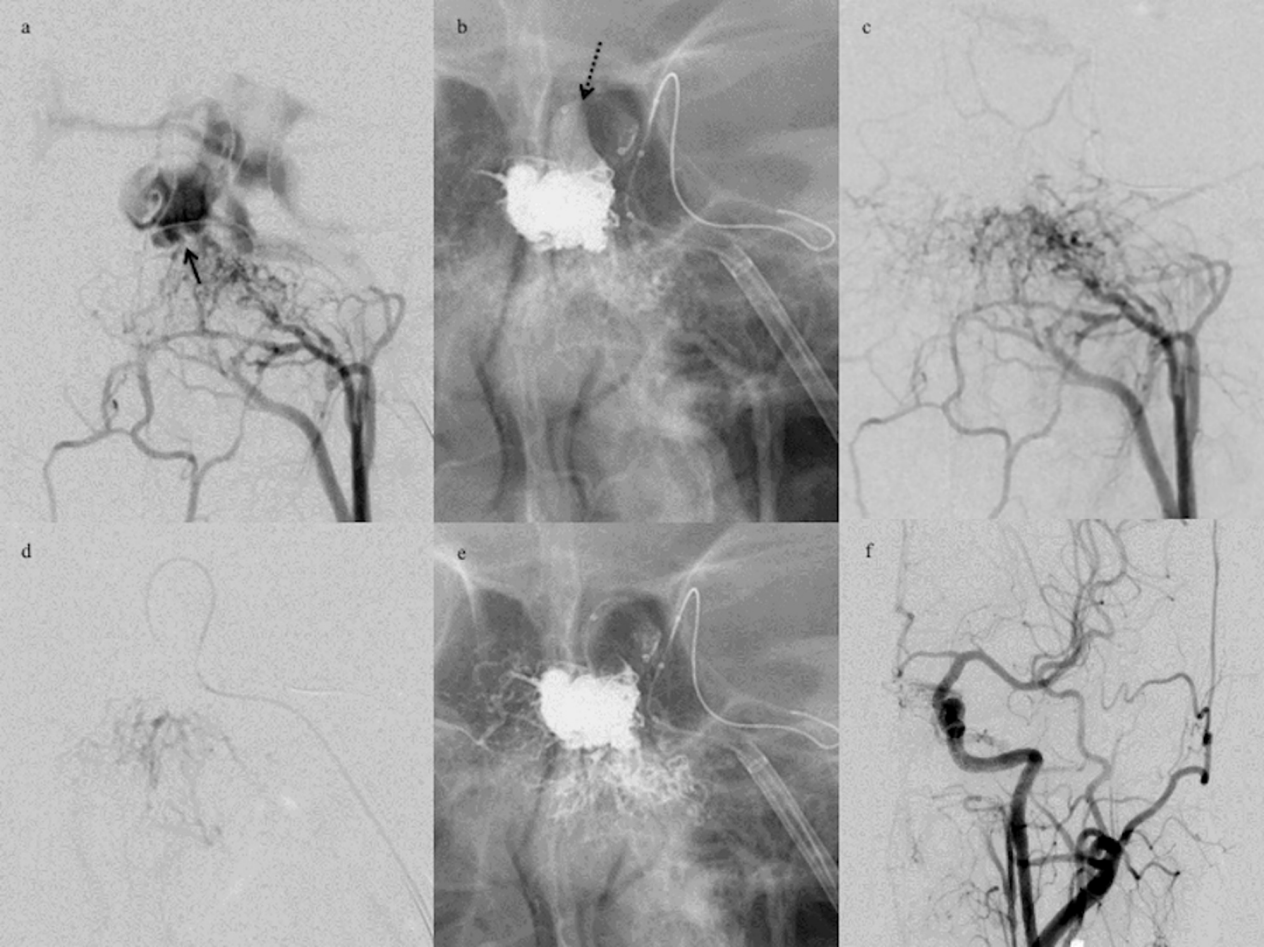
Illustrative case 2. A DLBM was used to identify the shunt point. Left ascending pharyngeal artery angiograms. (a) Anteroposterior view. Angiograms reveal the fistulous shunt in the posteromedial compartment of cavernous sinus (black arrows). (b) A non-subtracted image reveals DLBM inflation (dotted black arrow) after coil embolization of the shunt point. (c) An ascending pharyngeal artery angiogram during balloon inflation reveals occlusion of the residual fistula. (d) Injection from the microcatheter during microballoon inflation reveals the arteries feeding into the fistulous shunt. (e) A non-subtracted image depicts Onyx infiltration into the feeding artery. (f) A post-embolization CCAG reveals near-complete occlusion of the fistulous shunt. DLBM, dual-lumen balloon microcatheter; CCAG, common carotid artery angiogram

**Figure 3:**
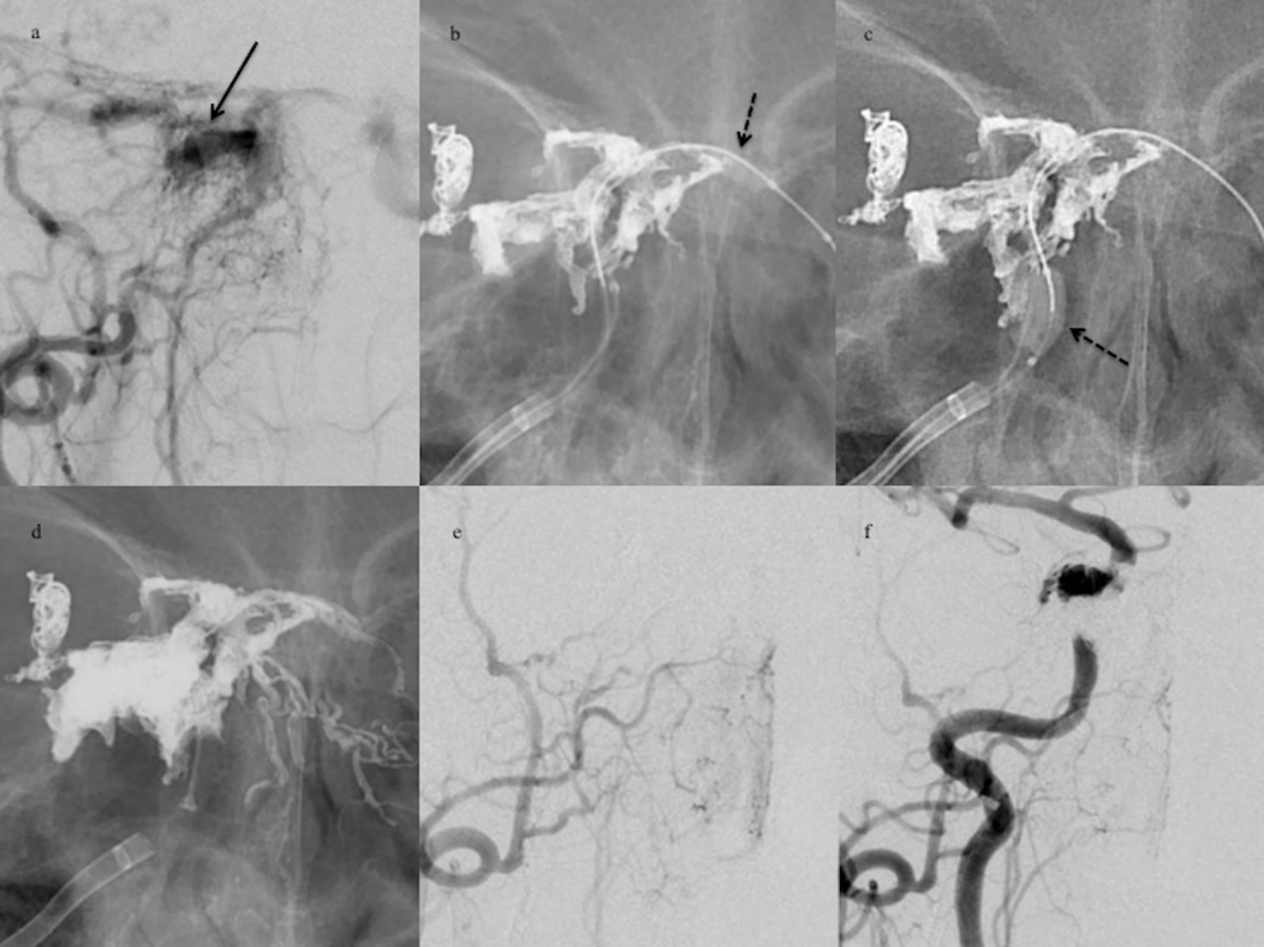
Illustrative case 3. A DLBM was used to prevent leakage to the intercavernous sinus and IPS. Right ECAG. (a) Anteroposterior view. Angiograms reveal the fistulous shunt in the posterolateral compartment of cavernous sinus (black arrows). (b) A non-subtracted image depicts DLBM inflation at the orifice of the intercavernous sinus (dotted black arrow) to protect against Onyx infiltration of the normal sinus. (c) A non-subtracted image depicts DLBM inflation at the inferior petrosal sinus (dotted black arrow) to protect against Onyx infiltration of the normal sinus. (d) Casts of Onyx and coils are visible at the end of the injection. (e) A post-embolization ECAG reveals non-opacification of the fistulous shunt. (f) A post-embolization CCAG reveals near-complete occlusion of the fistulous shunt. DLBM, dual-lumen balloon microcatheter; IPS, inferior petrosal sinus; ECAG, external carotid artery angiogram; CCAG, common carotid artery angiogram

We identified five patients to prevent leakage and two patients to indicate the shunt point (SP) (Table 1). Here, we describe the latter two illustrative cases, in which a DLBM was used to identify the SP (Figures 1, 2). We also describe an illustrative case, in which a DLBM was used to prevent leakage to the intercavernous sinus and IPS (Figure 3).

**Table 1:** Summary of the characteristics of patients and fistulas, treatment modalities, and dual-lumen microballoon use *At the 12-month follow-up. F, female; mRS, modified Rankin Scale; NBCA, n-butyl cyanoacrylate; IPS, inferior petrosal sinus; CO, complete occlusion

Case no.	Age (years), sex	mRS score before treatment	Presentation before treatment	Barrow type	Embolic materials	Balloon	Route	Complete occlusion at follow-up*	Complication	mRS score at follow-up*	Improvement of symptoms at follow-up*	Cranial nerve symptom at follow-up*	Main purpose
1	81, F	2	Left oculomotor and abducens nerve palsy	D	Coils + Onyx18	Scepter XC	IPS	CO	-	0	+	-	Identification of the shunt point
2	48, F	1	Tinnitus	D	Coils + Onyx18	Scepter XC	IPS	CO	-	0	+	-	Identification of the shunt point
3	63, F	2	Right oculomotor nerve palsy	D	Coils + Onyx18	Scepter XC	IPS	CO	-	1	+	Right oculomotor nerve palsy	Protection of normal venous drainage
4	80, F	1	Left chemosis and exophthalmos	D	Coils + Onyx18	Scepter XC	IPS	CO	-	0	+	-	Protection of normal venous drainage
5	58, F	1	Left abducens nerve palsy	D	Coils + NBCA + Onyx18	Scepter XC	IPS	CO	-	0	+	-	Protection of normal venous drainage
6	70, F	2	Right chemosis and exophthalmos	D	Coils + NBCA + Onyx18	Scepter XC	IPS	CO	-	0	+	-	Protection of normal venous drainage
7	73, F	1	Right oculomotor and abducens nerve palsy	D	Coils + NBCA + Onyx18	Scepter XC	IPS	CO	-	0	+	-	Protection of normal venous drainage

For control angiography, a 4-Fr catheter was introduced to the external or common carotid artery related to the CSDAVF from a femoral artery. The Flexor Shuttle® 7-Fr Guiding Sheath (Cook Medical, Bloomington, IN, USA) was placed into the internal jugular vein from a femoral vein. Next, a 6-Fr guiding catheter was placed into the IPS over a 0.035-inch outer diameter guidewire (Radifocus®, Terumo, Tokyo, Japan), and a DMSO-compatible microcatheter (Echelon™, Medtronic, Irvine, CA, USA) was advanced through the guiding catheter with a 0.014-inch guidewire (CHIKAI, Asahi Intecc, Aichi, Japan). A DLBM was then navigated to the SP of the cavernous sinus through the IPS. Once inflated, the point where the balloon appeared to occlude the fistula on angiography was considered the SP. Finally, we performed coil embolization of the SP to provide a scaffold for liquid materials, in which the liquid materials were injected into the SP and minimized the infiltration to the feeding arteries during DLBM inflation. Corticosteroids were administered for several days after the procedure. In the absence of complications, patients were extubated in the operating room and discharged after four to five days.

In two cases, DLBM successfully identified the SP without complications. Five patients were treated with DLBM, which prevented liquid material leakage to normal venous drainage. Patients who underwent TVE with a DLBM achieved complete occlusion at the follow-up. And one patient presented with ocular symptoms and cranial nerve palsy at the 12-month follow-up. However, all patients showed improved symptoms at the 12-month follow-up. Treatment-related complications were not identified.

## Discussion

We described the performance of TVE with a DLBM and liquid materials (NBCA and Onyx) for CSDAVF.

Coil packing of the entire cavernous sinus may induce sinus and ophthalmic vein thrombosis and aggravate symptoms. However, targeted CSDAVF embolization does not exacerbate symptoms because the target is limited to a small compartment of the cavernous sinus [8]. TVE with coils and liquid materials for CSDAVF resulted in higher complete occlusion rates without increasing complication rates [15]. This technique enables target embolization while placing coils as a scaffold for liquid materials and controls liquid material infiltration into the feeding arteries. These advantages increase curative rates associated with TVE.

Although TAE with Onyx is a safe and effective alternative for patients with CSDAVF experiencing failed transvenous catheterization, the use of liquid materials carries the risk of dangerous embolization of anastomoses between the dural branches of the external and internal carotid arteries, vasa nervorum, ophthalmic artery, or vertebral artery [10,11]. Therefore, the current indication of transarterial Onyx embolization for a CSDAVF is limited to patients who experience an unsuccessful TVE. The use of Onyx during targeted TVE for a CSDAVF does not require packing of the cavernous sinus with the embolic agent.

For a carotid-cavernous fistula (Barrow type A), intra-arterial balloon-assisted Onyx embolization is a powerful option that prevents inadvertent migration of the embolic material into the arterial system, facilitates visualization of the internal carotid artery, and provides a buttress for coils deployed in the cavernous sinus [16]. Contrast agent microinjected through an inflated dual-lumen balloon can characterize the carotid wall defect during embolization [17]. A compliant balloon might be navigated into the transverse sinus to protect against the reflux of Onyx to the normal sinus in the DAVF of the transverse-sigmoid sinus [13]. No newly occurring cranial nerve palsy was fortunately detected in any of our cases. However, Su et al. [18] reported that in a series of 121 patients treated for indirect carotid-cavernous fistula, the incidence of immediate post-treatment newly occurring cranial nerve palsy was 19.8% following TVE. Su et al. postulated that the presumptive causes included progressive thrombosis, mass effect due to embolization materials, and direct nerve injury by the microwire and microcatheter [18]. Thus, our use of angiography during Scepter XC balloon inflation to characterize the SP and Scepter XC to prevent liquid material leakage to the normal venous drainage might decrease the mass effect due to liquid materials, thereby minimizing the causes of newly occurring cranial nerve palsy. Therefore, a detailed understanding of the angiographical anatomy of cavernous sinus compartment is essential. However, in a TVE using liquid materials for CSDAVF, complications such as cranial nerve palsy and embolic agent migration into the internal carotid artery due to numerous anastomoses must be considered.

Our technique has some limitations. Although it is useful when the SP is limited to a small compartment, targeted TVE is difficult for a CSDAVF with multiple diffuse shunts. In such cases, TVE involves coil embolization of the cortical venous reflux and SP embolization with liquid embolic agents while protecting the normal venous drainage route. Additionally, DMSO-related toxicity may occur during TVE for CSDAVF treatment with Onyx. Development of trigeminocardiac reflex, inappropriate antidiuretic hormone syndrome, and acute respiratory distress syndrome are potential side effects of DMSO; therefore, slow injection is paramount for preventing toxicity [19,20]. Infiltration of liquid materials into the feeding artery should be controlled. Moreover, complications such as cranial nerve palsy and embolic agent migration into the internal carotid artery due to numerous anastomoses must be considered. The major drawback of our study is the low number of patients on whom our technique was applied (n = 7). Therefore, we aim to collect additional data and further summarize our experiences in a larger study.

## Conclusions

This report describes a challenging technique for target embolization with a DLBM and liquid materials for CSDAVF. The DLBM contributes to characterization of the SP for CSDAVF. No new cranial nerve palsy was luckily detected in our cases; thus, this technique might result in decreasing the mass effect of the cavernous sinus and may lead to useful TVE with liquid materials for CSDAVF in the future. However, there is generally a risk in using liquid materials in the cavernous sinus; therefore, further consideration is needed in the future.
